# 1-(4,6-Dimethyl­pyrimidin-2-yl)thio­urea

**DOI:** 10.1107/S1600536811048148

**Published:** 2011-11-19

**Authors:** Sohail Saeed, Naghmana Rashid, Jerry P. Jasinski, James A. Golen

**Affiliations:** a Department of Chemistry, Research Complex, Allama Iqbal Open University, Islamabad 44000, Pakistan; b Department of Chemistry, Keene State College, 229 Main Street, Keene, NH 03435-2001, USA

## Abstract

In the crystal structure of the title compound, C_7_H_10_N_4_S, weak inter­molecular N—H⋯S inter­actions form a two-dimensional network parallel to the *ab* plane. An intra­molecular N—H⋯N hydrogen bond occurs.

## Related literature

For structural characterization of *N*-substituted thio­urea derivatives with heterocyclic substituents, see: Saeed *et al.* (2010*a* ▶,*b* ▶, 2011 ▶). For standard bond lengths, see Allen *et al.* (1987 ▶).
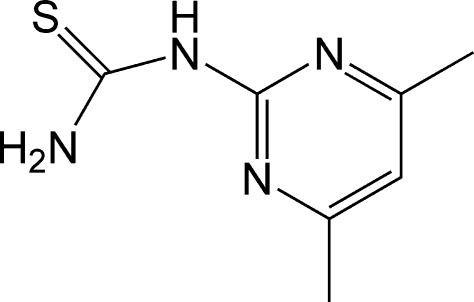


## Experimental

### 

#### Crystal data

C_7_H_10_N_4_S
*M* *_r_* = 182.25
Orthorhombic, 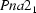

*a* = 8.3372 (5) Å
*b* = 15.8303 (10) Å
*c* = 6.618 (1) Å
*V* = 873.45 (15) Å^3^
*Z* = 4
Mo *K*α radiation
μ = 0.32 mm^−1^
*T* = 173 K
0.30 × 0.20 × 0.18 mm

#### Data collection

Oxford DiffractionXcalibur Eos Gemini diffractometer
Absorption correction: multi-scan (*CrysAlis RED*; Oxford Diffraction, 2010 ▶) *T*_min_ = 0.910, *T*_max_ = 0.945
7240 measured reflections
2057 independent reflections
1588 reflections with *I* > 2σ(*I*)
*R*_int_ = 0.043

#### Refinement

*R*[*F* ^2^ > 2σ(*F* ^2^)] = 0.053
*wR*(*F* ^2^) = 0.144
*S* = 1.10
2057 reflections
120 parameters
4 restraints
H atoms treated by a mixture of independent and constrained refinement
Δρ_max_ = 0.57 e Å^−3^
Δρ_min_ = −0.23 e Å^−3^

### 

Data collection: *CrysAlis PRO* (Oxford Diffraction, 2010 ▶); cell refinement: *CrysAlis PRO*; data reduction: *CrysAlis RED* (Oxford Diffraction, 2010 ▶); program(s) used to solve structure: *SHELXS97* (Sheldrick, 2008 ▶); program(s) used to refine structure: *SHELXL97* (Sheldrick, 2008 ▶); molecular graphics: *SHELXTL* (Sheldrick, 2008 ▶); software used to prepare material for publication: *SHELXTL*.

## Supplementary Material

Crystal structure: contains datablock(s) global, I. DOI: 10.1107/S1600536811048148/im2335sup1.cif

Structure factors: contains datablock(s) I. DOI: 10.1107/S1600536811048148/im2335Isup2.hkl

Supplementary material file. DOI: 10.1107/S1600536811048148/im2335Isup3.cml

Additional supplementary materials:  crystallographic information; 3D view; checkCIF report

## Figures and Tables

**Table 1:** Hydrogen-bond geometry (Å, °)

*D*—H⋯*A*	*D*—H	H⋯*A*	*D*⋯*A*	*D*—H⋯*A*
N1—H1*A*⋯N4	0.90 (2)	1.99 (3)	2.676 (3)	131 (3)
N1—H1*B*⋯S1^i^	0.87 (2)	2.58 (2)	3.399 (2)	159 (4)
N2—H2*A*⋯S1^ii^	0.85 (2)	2.53 (2)	3.338 (2)	160 (4)

Symmetry codes: (i) 
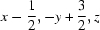
; (ii) 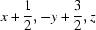.

**Fig. 1.**
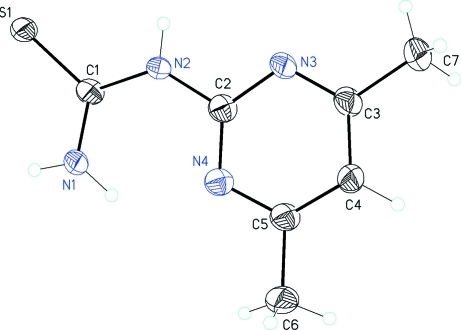
Molecular structure of the title compound showing the atom labeling scheme and 30% probability displacement ellipsoids.

**Fig. 2.**
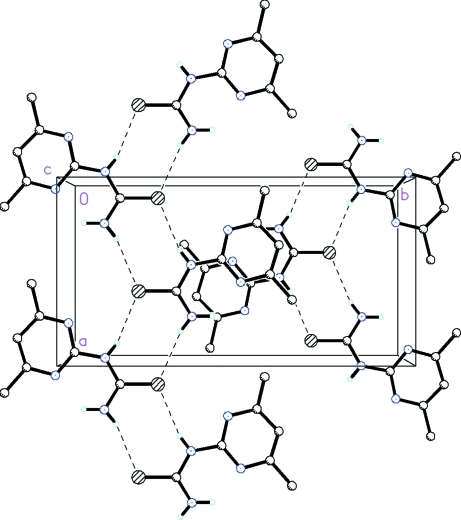
Packing diagram of the title compound viewed along the c axis. Dashed lines indicate weak N—H···S intermolecular interactions forming a 2-D network along [110].

## supplementary crystallographic information

### Comment

The crystal structure of the title compound is a byproduct of the reaction of 1-(4,6-dimethylpyrimidin-2-yl)-3-(3,5-dinitrophenyl)thiourea with a copper acetate salt. It is related to our previous studies on the structural chemistry of heterocyclic compounds containing an N-substituted thiourea (Saeed *et al.*, 2010*a*, 2010*b*, 2011). Herein, as a continuation of these studies, the structure of the title compound, (I), C_7_H_10_N_4_S, is described.

In the title compound, (I), (Fig. 1) the crystal packing is realized by intramolecular N1—H1···N4 hydrogen bonds and weak N—H···S intermolecular interactions (Table 1) forming a 2-D network along [110] (Fig. 2). Bond distances are in normal ranges (Allen *et al.* (1987).

### Experimental

After refluxing a reaction mixture of 1-(4,6-dimethylpyrimidin-2-yl)-3- (3,5-dinitrophenyl)thiourea with copper acetate salt, it was transfered into cold water. The crude solid product was filtered, washed again with water and purified by re-crystallization from ethanol (Yield: 45%). Single crystals of the title compound were obtained by recrystallisation from a dichloromethane/ethanol mixture (2:1).

### Refinement

H1A, H1B and H2A were located in a Fourier map and refined isotropically. All other H atoms were placed in their calculated positions and then refined using the riding model with atom—H bond lengths of 0.95Å (CH) or 0.98Å (CH_3_). Isotropic displacement parameters for these atoms were set to 1.19 (CH) or 1.48–1.50 (CH_3_) times *U*_eq_ of the parent atom. 928 Friedel pairs were measured.

### Crystal data

**Table:**

C_7_H_10_N_4_S	*F*(000) = 384
*M**_r_* = 182.25	*D*_x_ = 1.386 Mg m^−^^3^
Orthorhombic, *P**n**a*2_1_	Mo *K*α radiation, λ = 0.71073 Å
Hall symbol: P 2c -2n	Cell parameters from 2136 reflections
*a* = 8.3372 (5) Å	θ = 3.3–32.5°
*b* = 15.8303 (10) Å	µ = 0.32 mm^−^^1^
*c* = 6.618 (1) Å	*T* = 173 K
*V* = 873.45 (15) Å^3^	Block, pale yellow
*Z* = 4	0.30 × 0.20 × 0.18 mm

### Data collection

**Table:**

Oxford DiffractionXcalibur Eos Gemini diffractometer	2057 independent reflections
Radiation source: Enhance (Mo) X-ray Source	1588 reflections with *I* > 2σ(*I*)
graphite	*R*_int_ = 0.043
Detector resolution: 16.1500 pixels mm^-1^	θ_max_ = 27.9°, θ_min_ = 3.3°
ω scans	*h* = −10→10
Absorption correction: multi-scan (*CrysAlis RED*; Oxford Diffraction, 2010)	*k* = −20→20
*T*_min_ = 0.910, *T*_max_ = 0.945	*l* = −8→8
7240 measured reflections	

### Refinement

**Table:**

Refinement on *F*^2^	Primary atom site location: structure-invariant direct methods
Least-squares matrix: full	Secondary atom site location: difference Fourier map
*R*[*F*^2^ > 2σ(*F*^2^)] = 0.053	Hydrogen site location: inferred from neighbouring sites
*wR*(*F*^2^) = 0.144	H atoms treated by a mixture of independent and constrained refinement
*S* = 1.10	*w* = 1/[σ^2^(*F*_o_^2^) + (0.0705*P*)^2^ + 0.3395*P*] where *P* = (*F*_o_^2^ + 2*F*_c_^2^)/3
2057 reflections	(Δ/σ)_max_ = 0.021
120 parameters	Δρ_max_ = 0.57 e Å^−^^3^
4 restraints	Δρ_min_ = −0.23 e Å^−^^3^

### Special details

**Table:**

Geometry. All e.s.d.'s (except the e.s.d. in the dihedral angle between two l.s. planes) are estimated using the full covariance matrix. The cell e.s.d.'s are taken into account individually in the estimation of e.s.d.'s in distances, angles and torsion angles; correlations between e.s.d.'s in cell parameters are only used when they are defined by crystal symmetry. An approximate (isotropic) treatment of cell e.s.d.'s is used for estimating e.s.d.'s involving l.s. planes.
Refinement. Refinement of *F*^2^ against ALL reflections. The weighted *R*-factor *wR* and goodness of fit *S* are based on *F*^2^, conventional *R*-factors *R* are based on *F*, with *F* set to zero for negative *F*^2^. The threshold expression of *F*^2^ > σ(*F*^2^) is used only for calculating *R*-factors(gt) *etc*. and is not relevant to the choice of reflections for refinement. *R*-factors based on *F*^2^ are statistically about twice as large as those based on *F*, and *R*- factors based on ALL data will be even larger.

### Fractional atomic coordinates and isotropic or equivalent isotropic displacement parameters (Å^2^)

**Table:**

	*x*	*y*	*z*	*U*_iso_*/*U*_eq_	
S1	0.39504 (8)	0.77736 (4)	0.3627 (3)	0.0334 (2)	
N1	0.2593 (2)	0.62628 (14)	0.3662 (11)	0.0295 (5)	
H1A	0.261 (4)	0.5693 (11)	0.374 (13)	0.035*	
H1B	0.179 (3)	0.6589 (17)	0.338 (8)	0.035*	
N2	0.5367 (2)	0.62855 (12)	0.3683 (10)	0.0241 (5)	
H2A	0.615 (3)	0.6613 (16)	0.345 (9)	0.029*	
N3	0.7243 (2)	0.52446 (13)	0.3633 (9)	0.0309 (6)	
N4	0.4441 (3)	0.48807 (14)	0.3725 (8)	0.0263 (5)	
C1	0.3948 (3)	0.67031 (15)	0.3601 (11)	0.0246 (6)	
C2	0.5672 (3)	0.54221 (16)	0.3715 (9)	0.0265 (6)	
C3	0.7616 (3)	0.44226 (17)	0.3597 (12)	0.0310 (6)	
C4	0.6435 (3)	0.38055 (16)	0.3665 (14)	0.0318 (6)	
H4A	0.6711	0.3225	0.3786	0.038*	
C5	0.4852 (3)	0.40536 (17)	0.3554 (10)	0.0292 (7)	
C6	0.3496 (3)	0.34350 (17)	0.3676 (15)	0.0403 (8)	
H6A	0.2609	0.3629	0.2823	0.060*	
H6B	0.3130	0.3390	0.5079	0.060*	
H6C	0.3863	0.2881	0.3205	0.060*	
C7	0.9355 (3)	0.42072 (19)	0.3708 (14)	0.0413 (9)	
H7A	0.9991	0.4684	0.3206	0.062*	
H7B	0.9568	0.3707	0.2878	0.062*	
H7C	0.9648	0.4089	0.5114	0.062*	

### Atomic displacement parameters (Å^2^)

**Table:**

	*U*^11^	*U*^22^	*U*^33^	*U*^12^	*U*^13^	*U*^23^
S1	0.0193 (3)	0.0239 (3)	0.0570 (5)	0.0017 (2)	−0.0110 (6)	−0.0034 (11)
N1	0.0161 (10)	0.0256 (10)	0.0466 (15)	0.0014 (8)	−0.007 (3)	−0.008 (3)
N2	0.0173 (10)	0.0222 (10)	0.0328 (13)	−0.0013 (8)	−0.005 (3)	−0.005 (3)
N3	0.0206 (11)	0.0298 (12)	0.0424 (16)	0.0013 (8)	−0.015 (2)	−0.008 (3)
N4	0.0254 (11)	0.0278 (10)	0.0257 (14)	−0.0015 (8)	−0.006 (2)	0.003 (2)
C1	0.0197 (11)	0.0293 (12)	0.0248 (15)	0.0002 (9)	−0.012 (2)	−0.001 (3)
C2	0.0257 (13)	0.0284 (12)	0.0254 (16)	−0.0001 (9)	−0.008 (3)	0.003 (3)
C3	0.0247 (13)	0.0335 (14)	0.0347 (17)	0.0019 (10)	−0.009 (3)	−0.006 (3)
C4	0.0270 (13)	0.0255 (12)	0.0430 (17)	0.0021 (10)	0.007 (4)	0.007 (4)
C5	0.0258 (13)	0.0304 (13)	0.0315 (18)	−0.0027 (10)	−0.009 (2)	0.003 (3)
C6	0.0283 (14)	0.0319 (14)	0.061 (2)	−0.0073 (11)	−0.013 (4)	0.001 (5)
C7	0.0291 (14)	0.0356 (15)	0.059 (2)	0.0061 (12)	−0.014 (4)	−0.001 (4)

### Geometric parameters (Å, °)

**Table:**

S1—C1	1.695 (3)	C3—C4	1.388 (4)
N1—C1	1.328 (3)	C3—C7	1.491 (4)
N1—H1A	0.904 (17)	C4—C5	1.379 (4)
N1—H1B	0.869 (18)	C4—H4A	0.9500
N2—C1	1.356 (3)	C5—C6	1.497 (4)
N2—C2	1.390 (3)	C6—H6A	0.9800
N2—H2A	0.846 (17)	C6—H6B	0.9800
N3—C3	1.338 (3)	C6—H6C	0.9800
N3—C2	1.341 (3)	C7—H7A	0.9800
N4—C2	1.337 (3)	C7—H7B	0.9800
N4—C5	1.358 (3)	C7—H7C	0.9800
			
C1—N1—H1A	120.7 (19)	C5—C4—H4A	120.8
C1—N1—H1B	110 (2)	C3—C4—H4A	120.8
H1A—N1—H1B	128 (3)	N4—C5—C4	120.8 (3)
C1—N2—C2	129.7 (2)	N4—C5—C6	115.8 (3)
C1—N2—H2A	112 (2)	C4—C5—C6	122.2 (2)
C2—N2—H2A	118 (2)	C5—C6—H6A	109.5
C3—N3—C2	115.6 (2)	C5—C6—H6B	109.5
C2—N4—C5	115.1 (2)	H6A—C6—H6B	109.5
N1—C1—N2	119.0 (2)	C5—C6—H6C	109.5
N1—C1—S1	121.71 (19)	H6A—C6—H6C	109.5
N2—C1—S1	119.07 (17)	H6B—C6—H6C	109.5
N4—C2—N3	128.0 (2)	C3—C7—H7A	109.5
N4—C2—N2	119.3 (2)	C3—C7—H7B	109.5
N3—C2—N2	112.6 (2)	H7A—C7—H7B	109.5
N3—C3—C4	121.2 (2)	C3—C7—H7C	109.5
N3—C3—C7	116.6 (2)	H7A—C7—H7C	109.5
C4—C3—C7	121.8 (2)	H7B—C7—H7C	109.5
C5—C4—C3	118.5 (2)		
			
C2—N2—C1—N1	4.0 (12)	C2—N3—C3—C4	−0.7 (11)
C2—N2—C1—S1	178.8 (6)	C2—N3—C3—C7	−174.2 (6)
C5—N4—C2—N3	−2.7 (10)	N3—C3—C4—C5	6.9 (12)
C5—N4—C2—N2	173.7 (5)	C7—C3—C4—C5	180.0 (8)
C3—N3—C2—N4	−1.4 (10)	C2—N4—C5—C4	9.0 (11)
C3—N3—C2—N2	−178.1 (6)	C2—N4—C5—C6	176.8 (6)
C1—N2—C2—N4	−2.1 (11)	C3—C4—C5—N4	−11.2 (12)
C1—N2—C2—N3	174.9 (8)	C3—C4—C5—C6	−178.2 (8)

### Hydrogen-bond geometry (Å, °)

**Table:**

*D*—H···*A*	*D*—H	H···*A*	*D*···*A*	*D*—H···*A*
N1—H1A···N4	0.90 (2)	1.99 (3)	2.676 (3)	131 (3)
N1—H1B···S1^i^	0.87 (2)	2.58 (2)	3.399 (2)	159 (4)
N2—H2A···S1^ii^	0.85 (2)	2.53 (2)	3.338 (2)	160 (4)

Symmetry codes: (i) *x*−1/2, −*y*+3/2, *z*; (ii) *x*+1/2, −*y*+3/2, *z*.
